# Assessment of Biological Age With Conventional Ultrasound Imaging as an Alternative to X‐Ray—A Pilot Study in Youth Soccer

**DOI:** 10.1002/ejsc.12264

**Published:** 2025-02-05

**Authors:** Chantal Widmer, Jasmin D. Busch, Dennis‐Peter Born, Michael Romann

**Affiliations:** ^1^ Department for Elite Sport Swiss Federal Institute of Sport Magglingen Magglingen Switzerland; ^2^ Department of Health Sciences and Technology ETH Zurich Zurich Switzerland; ^3^ Faculty of Science and Medicine University of Fribourg Fribourg Switzerland; ^4^ Department of Diagnostic Interventional and Paediatric Radiology Inselspital University of Bern Bern Switzerland; ^5^ Section for High‐Performance Sports Swiss Swimming Federation Bern Switzerland

**Keywords:** biological age, ossification ratio, skeletal maturity, talent development, talent selection

## Abstract

The aim of the study was to evaluate conventional ultrasound (US) as a radiation‐free alternative to X‐ray for determining biological age (BA; indicated by skeletal age). BA, was determined in 24 healthy, male, elite youth soccer goalkeepers around peak height velocity (11–16 years of age) using both X‐ray and conventional US scans of the left hand. X‐ray scans were evaluated using the Tanner–Whitehouse 2 method. Conventional US scans served to determine BA via ossification ratios of 13 hand and wrist bones. The new conventional US method showed very strong correlation with X‐ray *r* = 0.90 (*p* < 0.05). However, the agreement for the difference in BA and CA, which accounts for age‐related variance, was classified poor (ICC = 0.48, *p* < 0.05). Additionally, linear regression analysis and the Bland–Altman plot suggested the presence of a systematic and proportional overestimation of BA in younger players and an underestimation of BA in older players. Furthermore, Cohen's kappa showed a moderate agreement between players' classification into maturity groups for the two assessment methods. In conclusion, our study has shown that using US‐derived ossification ratios did not deliver valid results compared to X‐ray when determining BA in youth soccer goalkeepers.

**Summary**

- Conventional US could offer a **practical, radiation‐free alternative** for estimating skeletal maturity in youth athletes, though further refinement is needed to address measurement biases and improve reliability for widespread sports applications.
- Conventional ultrasound (US) for biological age (BA) assessment in youth soccer goalkeepers demonstrated a very strong correlation with X‐ray‐derived BA (*r* = 0.90, *p* < 0.05), supporting its **potential for noninvasive talent evaluation.**
- However, the study underscores **limitations in using US for maturity‐based adjustments** and the next step for further development in order to remove the variability between the methods and avoid potential misclassification of maturity stages.

## 1 Introduction

Growth and maturity are complex processes, which must be considered during talent development in sports. Traditionally, chronological age (CA) is used to define training and competition groups. However, during puberty, biological age (BA; indicated by skeletal age) can differ by up to five years in children of the same CA, thus affecting the development of physical performance and psychosocial factors, such as leadership, responsibility and resilience (Mirwald et al. 2002; Malina et al. 2004a; Carling et al. 2009). For example, biologically older athletes generally show superior strength, speed (Lefevre et al. 1990), agility and endurance performances (Towlson et al. 2018; Malina et al. 2004b) compared to biologically younger athletes of the same CA (Malina et al. 2004a; Sierra‐Díaz et al. 2017). This leads to a greater selection and promotion of biologically older athletes in sports that emphasise physical fitness and involve body contact, that is, soccer. Particularly, playing positions that are affected by maturity‐related performance factors, that is, body height in soccer goalkeepers, are typically biased towards biologically older individuals (Malina et al. 2004a; Sierra‐Díaz et al. 2017). However, to create equal opportunities for goalkeepers and to avoid the loss of potential talents from the late developing group, athletes with the highest potential for success should be supported, rather than simply the most biologically developed athletes (Williams, Ford, and Drust 2020). Therefore, considering BA in training and competition is essential. Grouping athletes according to BA, so‐called bio‐banding, resulted in more equal competition, improved talent development and reduced injury risk (Malina et al. 2019). However, the validity and reliability of the methods used to determine BA are fundamental to ensure an effective application of such methods that are correct for the development stage. As such, an accurate and valid method that allows frequent and regular determination of BA in goalkeepers is required (Malina et al. 2019; Leyhr et al. 2020).

Methods for estimating BA can be divided into skeletal, somatic and sexual age. A common and well accepted method for estimating *skeletal age* with high validity is analysing X‐rays of left hand and wrist, using evaluation methods such as the Greulich–Pyle (GP) and Tanner–Whitehouse (TW) atlases (Tanner 1981; Wan et al. 2019). However, X‐rays are expensive, require specialised equipment and expose individuals to radiation. While magnetic resonance imaging (MRI) offers a radiation‐free alternative, its high costs and time consumption limit its practicality in sports (Dvorak 2009; Rüeger et al. 2022). *Somatic age* can be estimated by noninvasive methods, which estimate peak height velocity (PHV)—the highest growth rate in a child's stature—or percentage of adult height. For example, the Mirwald method estimates the age at which an individual will reach or has reached PHV using their weight, sitting and standing height. However, this method becomes particularly inaccurate for individuals advanced or delayed in maturity distant from their PHV (Mirwald et al. 2002; Malina et al. 2004a, 2021). Similarly, the widely used Khamis–Roche method predicts the final adult height based on height, weight and mid‐parent height. Since the Khamis–Roche method was developed using data from healthy Caucasian children, its limitations in accuracy within the Caucasian population are expected to be even larger within populations with different growth trajectories, such as South Asian children (Malina et al. 2019; Khamis et al. 1994). *Sexual age* is limited to the pubertal stage, as it is determined by secondary sexual characteristics, that is, breast, genitalia and pubic hair development. However, both somatic and sexual age estimations are assessed through secondary indicators rather than directly examining skeletal structures, which can lead to overestimations or underestimations of skeletal age (Mirwald et al. 2002; Malina et al. 2004a).

The use of conventional and quantitative ultrasound (US) offers an alternative method to estimate skeletal age, with numerous advantages over alternative methods: it is nonionising, noninvasive, inexpensive and easy to use. Thus, US could be of particular interest for regular use in talent selection and development in sports (Rüeger et al. 2022; Cumming et al. 2024). While conventional US imaging utilises linear high‐frequency transducers to visualise bone structures such as ossification centres, the interpretation of the images can be affected by artefacts and low resolution at increasing depths. Quantitative US‐based methods, such as BAUS (SonicBone), measure the interaction between ultrasound waves and biological tissue (Cumming et al. 2024; Rachmiel et al. 2017) by utilising the differing sound velocities and distance attenuation factors of the bone and cartilage, but overlying soft tissues can affect reliability (Rüeger et al. 2022). Previous studies (Cumming et al. 2024; Utczas et al. 2017) have demonstrated the effectiveness of both conventional and quantitative US in assessing BA. Several studies have demonstrated promising results, indicating strong correlations between estimates of BA derived from quantitative US and X‐ray (Cumming et al. 2024; Utczas et al. 2017). However, Bland–Altman analyses indicated a variability in the measurements by showing a relatively large 95% limits of agreement (2.5 years) (Cumming et al. 2024). Similarly, the comparison of BA determined with MRI and quantitative US in elite youth soccer players not only showed strong correlations but also a substantial difference between the upper and lower limits of agreement (LoA) (3.93 years), which further highlights the variability between the methods (Leyhr et al. 2020). Additionally, a scoring system established for conventional US based on the TW method has shown very strong correlations with BA derived from X‐ray assessments, although the studies did include nonhealthy participants (Wan et al. 2019, 2021).

An accurate, pragmatic and noninvasive method for determining BA is particularly important for playing positions involving maturity‐related characteristics, such as height, play a crucial role in performance, including soccer goalkeepers (Sierra‐Díaz et al. 2017; Leyhr et al. 2020). Since there is there is no literature on the use of conventional US for BA estimation, data on the validity, accuracy, precision and reliability of conventional US‐based methods are required (Hopkins 1997; Kottner et al. 2011). Thus, the aim of this pilot study is (1) to evaluate the correlations between BA, maturity scores and BA‐CA derived from X‐ray and conventional US to determine their alignment, consistency and reliability. The hypotheses were that X‐ray and US‐derived scores would show strong correlations, indicating overall consistency, though variability may be observed in specific measures such as BA‐CA. In addition, BA derived from US is expected to (2) show a significant linear relationship with BA derived from X‐ray, supporting the predictive capability of conventional US for age estimation. Furthermore, Bland–Altman analysis is (3) expected to demonstrate strong agreement between BA determined with conventional US and X‐ray, whereas Cohen's kappa is expected to show almost perfect agreement in maturity score.

## 2 Materials and Methods

### 2.1 Study Population

A total of 24 healthy, male, elite youth soccer goalkeepers aged 11–16 years (13.29 ± 0.98 years) were recruited. The age of the participants was chosen close to PHV, as this is when the greatest difference and variability in BA occur (Malina et al. 2021). Written informed consent was obtained from participants' parents or guardians. Personal information was coded to guarantee data protection. This study was approved by the Swiss ethics committee (Nr. 2023‐01536) and was conducted in accordance with the Declaration of Helsinki. The exclusion criteria included acute trauma, musculoskeletal disorders in the left hand such as previous or current fractures and any growth or neurological disorders.

### 2.2 Measures

**FIGURE A1:**
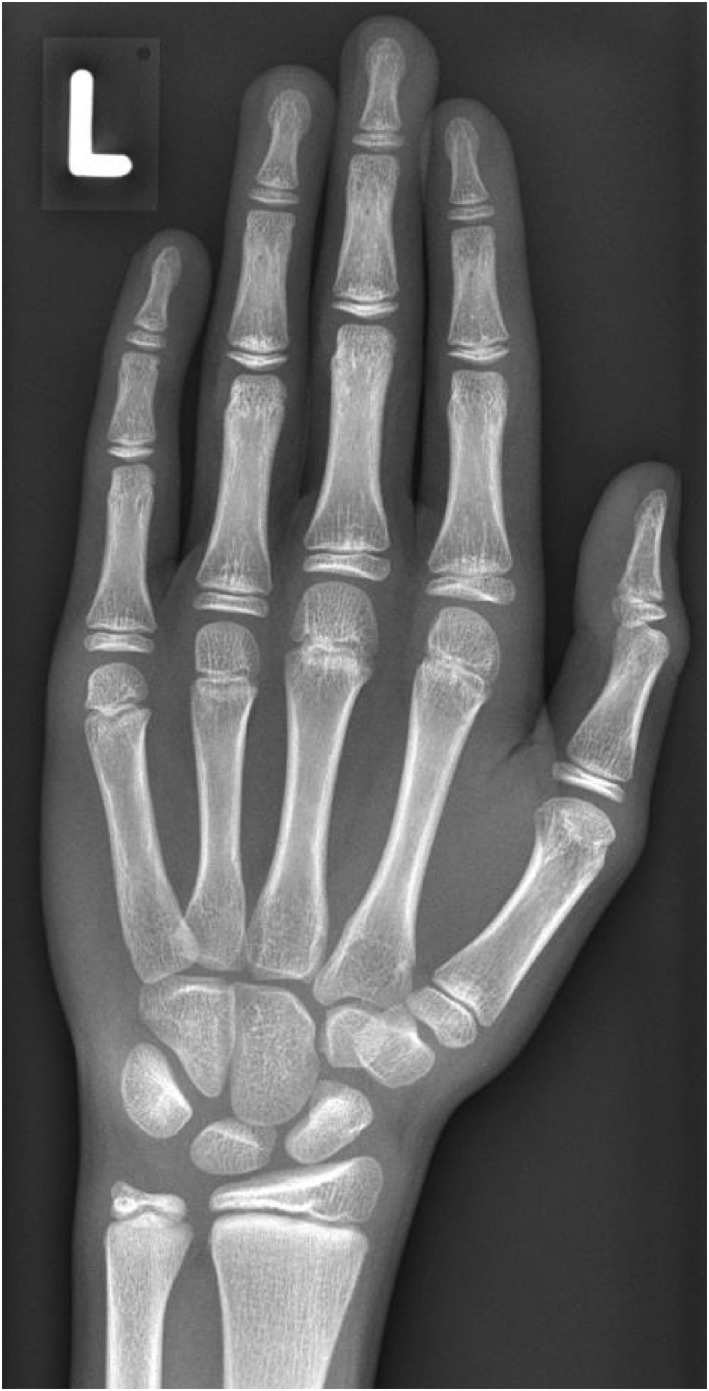
Left hand‐wrist X‐ray of participant eight.

Each participant underwent an X‐ray and conventional US scan of the left hand at the Institute for Diagnostic, Interventional and Paediatric Radiology at the Inselspital Bern. *X‐ray* examinations were performed by a professional radiologist on a commercial X‐ray device (PHILIPS DigitialDiagnost C90). The X‐ray was taken according to TW2 standards with a tube‐film distance of 120 cm, tube voltage of 40 kV and 2.0 mAs and a radiation time of 10 ms (Tanner 1983) (see appendix, Figure A1).

**FIGURE A2:**
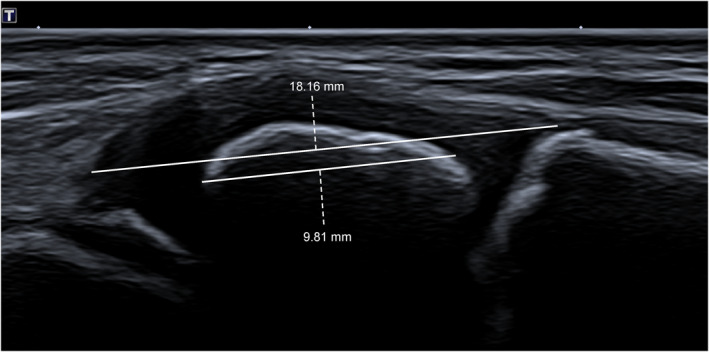
Ultrasound scan of the radius (participant 8) with the diameter of the ossification centre (= 9.81 mm) and diameter of the epiphysis (= 18.16 mm), resulting in an ossification ratio of 0.54.

**FIGURE A3:**
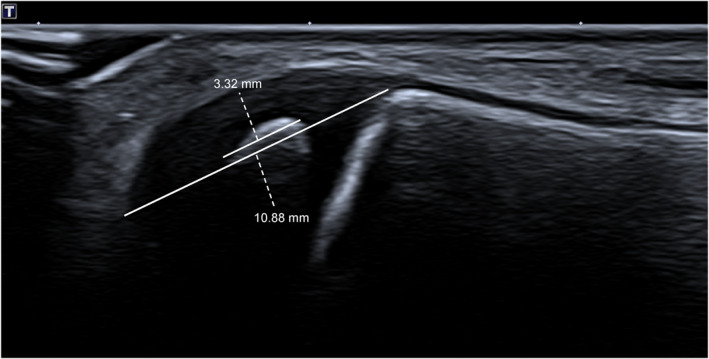
Ultrasound scan of ulna (participant 8) with the diameter of the ossification centre (= 3.32 mm) and diameter of the epiphysis (= 10.88 mm), resulting in an ossification ratio of 0.31.

*Conventional US* examinations were conducted by a professional radiologist on the same day as the X‐ray using a commercial sonographic machine (CANON Aplioi800) with a linear transducer (i24LX8). Scan parameters were as follows: 19 mm penetration (PEN), 1.75 mm penetration depth, 20 MHz frequency and 70 dB dynamic range (DR). The radius and ulna were scanned in a coronal plane, whereas metacarpals and phalanxes were scanned in a sagittal plane, according to Wan et al. (Wan et al. 2019) (see appendix, Figures A2 and A3). The same scanning order was followed for each site, starting with the radius, followed by the ulna, the first, third and fifth metacarpals and finally the phalanxes from proximal to distal.

### 2.3 Data Analysis

For each participant, CA was calculated as the difference between their date of birth and the day that X‐ray and US images were performed.

The *X‐ray* images were interpreted using the TW2 method. The TW2 maturity score was calculated and used to determine BA using the TW2 tables as previously described (Tanner 1983; Malina et al. 2018).

For the conventional *US* scans, BA was determined according to Wan et al. (Wan et al. 2019). First, the ossification ratio (OssR) of the distal radius and ulna; the first, third and fifth metacarpals; and phalanx were determined. The OssR was defined as the ratio of the diameter of the ossification centre to that of the epiphysis, with values ranging from 0% (no ossification centre) to 100% (complete fusion of the ossification centre) (Wan et al. 2019). Then, the skeletal maturity score (SMS) was calculated from the OssR of the 13 examined bones using the following formula in which OssR is the ossification ratio and W the weight coefficient of the 13 bones:

(1) $$SMS=\sum_{i=1}^{13}100\times {OssR}_{i}\times W_{i}$$

Due to different maturation rates, each bone was weighted based on the TW2 and as follows (Wan et al. 2019): 2 for the radius and ulna; 0.67 for the first metacarpal, the first proximal and distal phalanx; and 0.5 for the third and fifth metacarpal, third and fifth proximal, as well as the intermediate and distal phalanx. The resulting SMS was compared according to the TW2 maturity score and then converted to BA using the same tables as the TW2 method.

Two raters independently assessed all participants' X‐ray images to evaluate interrater reliability and agreement. The following classification was used to interpret the intraclass correlation coefficient (ICC): > 0.90 excellent reliability; 0.75–0.90 good reliability; 0.50–0.75 moderate reliability; and < 0.5 poor reliability (Koo et al. 2016). The interrater reliability for X‐ray‐derived BA assessments was excellent (ICC = 0.99, 95% confidence interval [0.99, 1], *p* < 0.05). Specifically, the mean interrater difference was 0.08 years with an SEE of 0.02 years. Given this high reliability, the mean value of the two raters was used for further analyses. The conventional US scans were analysed by one of the raters. All raters were blinded to the subjects' characteristics.

### 2.4 Statistical Analysis

All statistical analyses were conducted using *R* (Version 2022.07.1 build 554), with a significance level set at *p* < 0.05. The dataset was first examined to ensure it met key assumptions of normal distribution, linearity and homoscedasticity (consistent variability). The Shapiro–Wilk test (Shapiro et al. 1965) indicated that the data followed normal distribution, as the large *p*‐value suggested no significant deviation from normality. Scatter plots were used to assess linearity and homoscedasticity, suggesting these assumptions were met. Additionally, Q‐Q plots showed no significant outliers.

#### 2.4.1 Pearson's Correlation and Intraclass Correlation Coefficient

Pearson's correlation coefficient (*r*) was used to evaluate the relationship between several variables. This included examining the correlation between X‐ray TW2 maturity score and US SMS in order to compare the consistency of skeletal maturity scores derived from both methods. Additionally, the correlation between X‐ray‐derived BA and US‐derived BA was calculated to determine the level of linearity between these two methods, which is critical for practical applications such as bio‐banding or player‐labelling. Furthermore, the correlation between X‐ray derived BA and US SMS as comparison to Wan et al.‘s (Wan et al. 2019) was calculated. To determine bone‐specific variations, the correlation between OssR of each bone and X‐ray derived BA was examined. Pearson's correlations were defined as poor (*r* < 0.30), fair (*r* = 0.30–0.59), moderately strong (*r* = 0.6–0.79) and very strong (*r* ≥ 0.8) (Akoglu 2018). To evaluate consistency and agreement, ICC was calculated for the differences between BA and CA (BA‐CA) derived from X‐ray and US, respectively. This analysis provides insights into the reliability and agreement of these methods when measuring age differences.

#### 2.4.2 Linear Regression Analysis

Linear regression analysis was performed to assess the relationship between BA derived from X‐ray (independent variable) and BA derived from US (dependent variable), providing insights into the linearity of the relationship between the two methods.

#### 2.4.3 Agreement

The agreement between X‐ray‐ and US‐derived BA was analysed using the Bland–Altman plot (Hopkins 1997; Bland et al. 1999) and a one‐sampled *t*‐test against zero. The mean, the mean difference in years, standard deviation (SD) of mean difference, 95% limits of agreement (LoA) and standard error of estimate (SEE) were calculated for BA. The difference between BA derived from X‐ray compared to US assessment was plotted against the average of both methods.

Additionally, Cohen's kappa coefficient *(k)* (Kottner et al. 2011; Cohen 1960) was used to determine the agreement between participant classification into ‘early’, ‘on‐time’ and ‘late’ maturing by X‐ray compared to US based on their differences between BA and CA. Following previous studies (Malina et al. 2004a), participants were classified as ‘early’ if their BA was more than one year ahead of their CA (BA‐CA > 1), ‘on‐time’ if their BA was within one year of their CA (1 ≥ (BA‐CA) ≥ −1) and ‘late’ if their BA was more than one year behind their CA (BA‐CA < −1). Cohen's kappa results were interpreted as follows: *k* ≤ 0 indicated no agreement, 0.01–0.20 as none to slight, 0.21–0.40 as fair, 0.41–0.60 as moderate, 0.61–0.80 as substantial and *k* ≥ 0.81 as almost a perfect agreement (Kottner et al. 2011; Cohen 1960).

## 3 Results

The mean CA was 13.29 ± 0.98 years of age. The mean BAs were 14.33 ± 1.27 years and 14.99 ± 0.54 years when determined by X‐ray and US, respectively.

Table 1 shows the OssR values for each bone, ordered by US‐determined BA. With increasing BA, there was no consistent increase in the OssR across any of the 13 bones measured. One participant reached full ossification in the third and fifth distal phalanx, as well as the fifth intermediate phalanx, at a US‐determined BA of 15.9 years.

**TABLE 1:** Ossification ratios for each bone, ordered by biological age from the conventional ultrasound. Values are given as the mean.

BA	N	Radius	Ulna	MC1	PP1	DP1	MC3	PP3	IP3	DP3	MC5	PP5	IP5	DP5
13.6	1	0.49	0.25	0.57	0.32	0.50	0.76	0.48	0.38	0.45	0.67	0.49	0.31	0.48
14.0	1	0.54	0.30	0.52	0.36	0.77	0.77	0.41	0.39	0.57	0.59	0.25	0.45	0.52
14.4	2	0.55	0.39	0.52	0.44	0.61	0.81	0.51	0.50	0.53	0.75	0.45	0.37	0.53
14.6	1	0.52	0.51	0.73	0.43	0.65	0.94	0.37	0.42	0.52	0.57	0.50	0.41	0.63
14.7	2	0.55	0.38	0.82	0.45	0.59	0.92	0.53	0.50	0.46	0.77	0.53	0.46	0.62
14.8	1	0.62	0.35	0.57	0.44	0.75	0.85	0.52	0.54	0.52	0.92	0.47	0.59	0.76
14.9	2	0.64	0.39	0.82	0.50	0.58	0.90	0.54	0.45	0.50	0.91	0.57	0.45	0.58
15.0	2	0.55	0.53	0.71	0.53	0.68	0.83	0.54	0.58	0.52	0.77	0.55	0.59	0.66
15.1	2	0.67	0.39	0.84	0.61	0.57	0.94	0.61	0.55	0.59	0.85	0.58	0.42	0.63
15.2	2	0.59	0.54	0.85	0.51	0.84	0.93	0.55	0.58	0.53	0.87	0.46	0.51	0.60
15.3	4	0.70	0.54	0.67	0.65	0.66	0.84	0.57	0.61	0.57	0.81	0.63	0.51	0.59
15.6	1	0.78	0.50	0.73	0.83	0.75	0.89	0.71	0.67	0.59	0.98	0.65	0.49	0.57
15.7	2	0.75	0.69	0.64	0.70	0.65	0.86	0.69	0.66	0.60	0.88	0.65	0.50	0.64
15.9	1	0.78	0.61	0.82	0.76	0.29	0.75	0.64	0.68	1.00	0.87	0.79	1.00	1.00

Abbreviations: BA, biological age determined by ultrasound; DP1, first distal phalanx; DP3, third distal phalanx; DP5, fifth distal phalanx; IP3, third intermediate phalanx; IP5, fifth intermediate phalanx; MC1, first metacarpal; MC3, third metacarpal; MC5, fifth metacarpal; N, Number of participants; PP1, first proximal phalanx; PP3, third proximal phalanx; PP5, fifth proximal phalanx.

Both the correlations between the SMS and TW2 maturity score (*r* = 0.87, *p* < 0.05), as well as the correlation between SMS and X‐ray derived BA (*r* = 0.89, *p* < 0.05), indicated a strong relationship, whereas the correlation between BA derived from US and X‐ray (*r* = 0.90, *p* < 0.05) was very strong. Correlation values between OssR of each bone with the X‐ray‐derived BA ranged from −0.02 to 0.87 (Table 2). Negative correlations were observed in the third metacarpal and the first distal phalanx, whereas the other bones displayed positive correlations.

**TABLE 2:** Pearson's correlation coefficient (*r*) between biological age assessed by X‐ray and ossification ratio of the 13 bones assessed by conventional ultrasound.

	Radius	Ulna	MC1	PP1	DP1	MC3	PP3	IP3	DP3	MC5	PP5	IP5	DP5
OssR	0.78	0.70	0.14	0.87	−0.02	−0.02	0.69	0.70	0.57	0.46	0.52	0.65	0.58

Abbreviations: DP1, first distal phalanx; DP3, third distal phalanx; DP5, fifth distal phalanx; IP3, third intermediate phalanx; IP5, fifth intermediate phalanx; MC1, first metacarpal; MC3, third metacarpal; MC5, fifth metacarpal; OssR, Ossification Ratio; PP1, first proximal phalanx; PP3, third proximal phalanx; PP5, fifth proximal phalanx.

**FIGURE 1:**
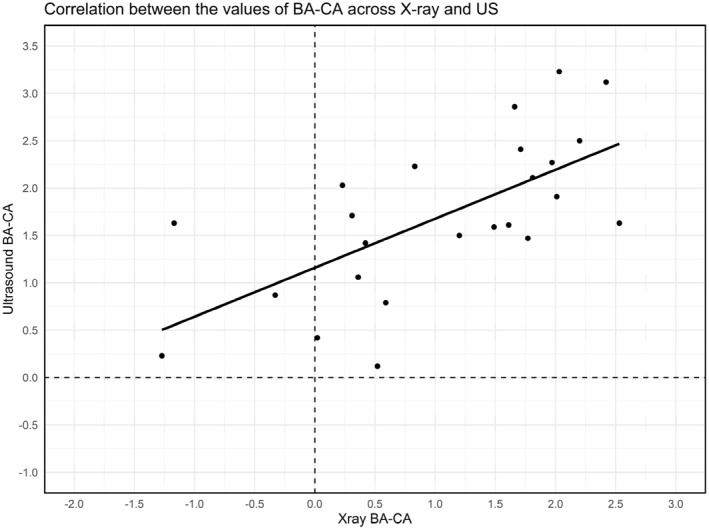
Scatter plot of the intraclass correlation for the difference in biological age and chronological age (BA‐CA) assessed by X‐ray and conventional US, with a trend line illustrating the relationship between the methods.

The ICC analysis was performed to examine consistency and agreement between the difference of BA and CA (BA‐CA) derived from X‐ray and US, respectively. The correlations for BA‐CA indicated poor agreement (ICC = 0.48, 95% confidence interval [0.11, 0.74], *p* < 0.05) between the two methods. Figure 1 shows a scatterplot of the correlation between X‐ray and US for BA‐CA.

**FIGURE 2:**
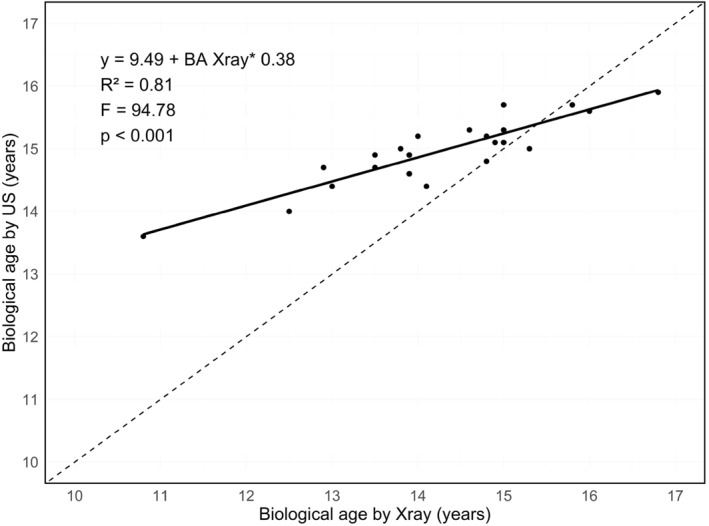
Biological age assessed by X‐ray and conventional ultrasound scans, with the line of identity (dashed line), the linear regression line (solid line) and the linear regression equation.

Figure 2 shows the linear regression between US and X‐ray‐derived BA against the line of identity, with an intercept of 9.49 years. The value of the slope was 0.38 and the standard error was 0.04. The model explained 81% of the variance in US‐derived BA (*R* ^2^ = 0.81). The F‐value was F_(1,22)_ = 94.78 and statistically significant (*p* < 0.001).

**FIGURE 3:**
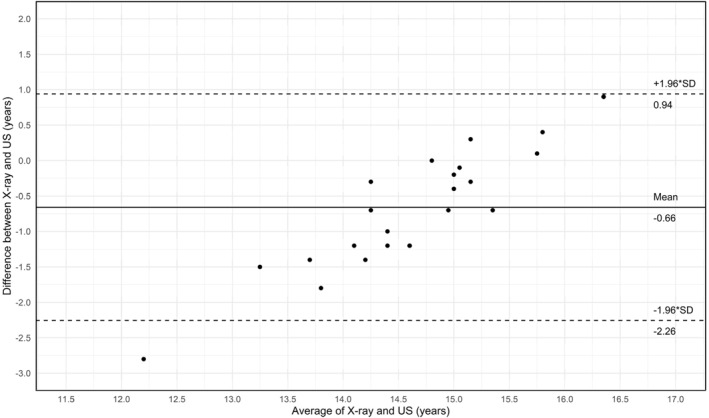
Bland–Altman plot representing biological age (in years) derived from X‐ray and conventional ultrasound. The solid line indicates the mean difference line, and the dotted lines represent the 95% of agreement (± 1.96 times the standard deviation).

The Bland–Altman plot (Figure 3) shows the agreement of BA between the two methods with the mean difference and LoA. The mean of both techniques was 14.66 ± 1.02 years. The mean difference was −0.66 years (95% confidence interval [−1.00, −0.31], *p* < 0.05) and an SEE of 0.17 years. SEEs for BA were 0.26 and 0.11 years for X‐ray and US, respectively. The 95% LoA were ± 1.60 years.

Thirteen goalkeepers were classified as early, 9 as normal and 2 as late maturing using the X‐ray data. Using the US data, 19 goalkeepers were classified as early, 5 as normal and zero as late maturing. Cohen's Kappa coefficient was statistically significant (*p* < 0.05) and showed moderate agreement (*k* = 0.41).

## 4 Discussion

### 4.1 Overview and Key Findings

This pilot study aimed to evaluate the validity of using conventional US as a radiation‐free alternative to X‐ray for determining BA in youth soccer goalkeepers. X‐ray‐derived scores (TW2 and BA) and US‐derived scores (SMS and BA) showed strong to very strong correlations. However, the agreement between the two methods, assessed by ICC, linear regression analysis and the Bland–Altman plot was poor, and Cohen's kappa showed only a moderate agreement between players' classification into maturity groups for the two assessment methods. As such, these results suggest that US‐derived OssR is not a valid alternative to BA assessments performed by X‐ray in youth soccer goalkeepers.

### 4.2 Intraclass Correlation Coefficient

The strong and very strong correlations between X‐ray and US measures of maturity align with the previous research on BA determination with conventional US (Wan et al. 2019; Lv et al. 2023). However, correlations solely indicate the degree to which the variables change together (i.e., their association) and do not inform on agreement or reliability of a certain measure. In addition, including participants from a broad age range, as in this study, can result in higher correlation coefficients compared to narrower age groups (Cumming et al. 2024; Rachmiel et al. 2017). As the ICC score for the difference in BA and CA (BA‐CA) between the two methods considers age‐related variance, it represents a more robust assessment of reliability and agreement of US‐derived BA. While previous studies have shown very strong correlations (ICC = 0.93) between quantitative US (BAUS) and X‐ray for BA determination (Cumming et al. 2024), the present results with conventional US revealed a poor agreement between the two methods (ICC = 0.48). This discrepancy between the results of quantitative and conventional US could be influenced by over‐ and underestimation of BA of youngest and oldest athletes of the sample. For instance, the US‐derived OssR may overestimate the maturation status in younger athletes, as the incomplete ossification process often results in a poorly defined ossification centre. Boundaries of the ossification centre may appear larger on US images due to factors such as partial ossification or the inclusion of the surrounding unossified tissue. In contrast, the maturation status of older athletes may be underestimated due to the near‐complete fusion of the ossification centre, which diminishes the visibility of the fusion line in the US images. This can lead to lower ICC scores, indicating a poor agreement. However, these rather practical observations and experience from our analyses need further elevation in future studies.

### 4.3 Systematic and Proportional Bias: Implications for Practical Use

**FIGURE A4:**
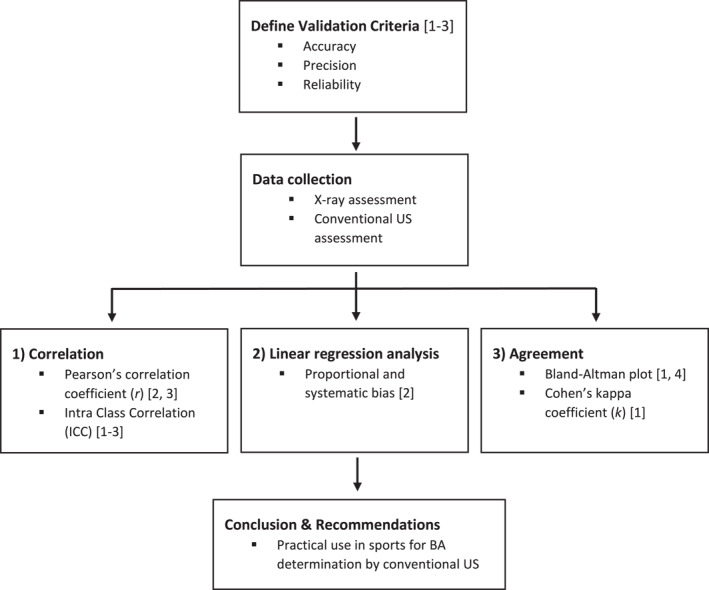
Validation process of a conventional ultrasound for determining biological age based on accuracy, precision and reliability. Key analyses include correlation, linear regression analysis and agreement measures.

Additionally, linear regression analysis showed a deviation from the line of identity when comparing BA derived from X‐ray (independent variable) with BA derived from US (dependent variable). The positive gradient of 0.38 in the regression suggests a proportional bias between the two methods, that is, the difference between the methods systematically changes with increased BA. This aligns with the results of the Bland–Altman plot, where this proportional bias between the two methods is highlighted by the positive trend in scatter points around the mean difference line. This proportional bias implies a systematic error and a lack of accuracy for certain age groups, as conventional US overestimates BA in younger participants and underestimates BA in older participants. Furthermore, the intercept of 9.49 years shown in the linear regression analysis differs from zero and indicates a systematic bias, that is, a consistent difference in BA measurements between the two methods. This systematic bias is also evident in the analysis of the Bland–Altman plot, where the mean difference of −0.66 years indicates that, on average, estimated BA was lower when using X‐ray compared to US However, introducing a correction factor could mitigate this systematic bias. Previous studies indicate higher precision and accuracy of US‐determined BA (compared to X‐ray) with conventional US (as used in the present study) resulting in no or positive bias (Wan et al. 2019, 2021; Lv et al. 2023), whereas quantitative US resulted in a negative bias (Cumming et al. 2024; Utczas et al. 2017). These differences in BA accuracy may be due to differences in comparison methods or participant characteristics. Specifically, Utczas et al. (Utczas et al. 2017) and Wan et al. (Wan et al. 2019) used the TW3 or GP method as a US comparison. It is however well documented, that different methods of interpreting X‐rays can influence BA measures. For example, the TW3 method is known to overestimate BA by 0.5 years (Malina et al. 2018). Other studies included nonhealthy children form a different ethnic group. These differences in population may have influenced BA, and as such, may explain the difference in outcomes (Wan et al. 2019, 2021). For US to become a useful alternative in sports setting to existing methods like the TW3 method, it needs to show improved accuracy, precision, reliability and practicality for field use (Lv et al. 2023) (see appendix, Figure A4).

Furthermore, the difference between the upper and lower 95% LoA in the Bland–Altman plot of this study was 3.20 years. This is higher than the 1.71–2.85 years observed in previous studies using conventional US (Wan et al. 2021; Lv et al. 2023). However, it falls within the range of 2.55–5.64 years observed in studies using quantitative US (Cumming et al. 2024; Rachmiel et al. 2017; Utczas et al. 2017). Similarly, Lehyr et al. (Leyhr et al. 2020) reported a wide LoA of 3.93 years in their comparison of MRI with quantitative US for BA assessments, despite high correlations. These findings highlight the variability in precision across different US methods and suggest that the precision of conventional US in our study is lower than previous studies using a similar technology. Additionally, in the context of bio‐banding, which groups athletes based on their BA rather than CA, this age range of 3.20 years would lead to inappropriate grouping and negate the purpose of creating an equal playing field (Towlson et al. 2018; Malina et al. 2019; Cumming et al. 2017). Similarly, player‐labelling, which categorises athletes based on their BA (Lüdin et al. 2022a), could result in significant misjudgements if based on US‐determined BA. Over‐ or underestimating their BA, and thus their potential, by up to 3.20 years, could lead to players being miscategorised, resulting in misguided trainings, missed opportunities and unfair comparison among peers (Cumming et al. 2017; Lüdin et al. 2022b).

This variability in BA determination also impacts frameworks like maturity‐based corrective adjustment procedures (Mat‐CAPs), which rely on accurate maturity assessments to correct performance scores (Charbonnet et al. 2022; Abbott et al. 2021). The wide range of 3.20 years observed in our study highlights reduced accuracy and precision in BA determination, potentially compromising the reliability of corrected scores, introducing bias and diminishing their effectiveness in talent identification. This inconsistency highlights the need for more accurate BA measurements to ensure fair and effective talent identification and development. Supporting this, Lehyr et al. (Leyhr et al. 2020), who compared MRI with quantitative US, Khamis–Roche and percentage of adult height, recommended using at least two methods to improve reliability, as a single method is not precise enough at the individual level.

### 4.4 Classification of Goalkeepers

Cohen's Kappa coefficient showed moderate agreement (*k* = 0.41) in classifying goalkeepers as early, normal and late maturing, which corresponds to previous research. However, there is a lack of agreement in classifying athletes' maturity status, as different methods do not categorise the same BA. When assessing maturity status, it is crucial to recognise the variability and measurement errors of each classification system and to interpret differences in maturity accordingly (Ruf et al. 2021; Malina et al. 2012). Therefore, further studies are needed.

### 4.5 Bone‐Specific Variability

Furthermore, the correlation between each bone's OssR and X‐ray‐derived BA showed a bone‐dependent variation, ranging from −0.02 to 0.87. In comparison, Wan et al. (Wan et al. 2019) reported a minimal and maximal *r* of 0.77 and 0.93, respectively. Negative correlations were observed in the third metacarpal and the first distal phalanx, whereas the other bones displayed positive correlations. None of the bones showed a continuous increase in OssR with higher BA, which was unexpected due to the age‐induced fusion of the bone and suggests that OssR may not adequately capture the progression of ossification and should therefore be further investigated and adapted. One potential improvement could be to use the maximal distance between the epiphysis and the metaphysis instead of the OssR.

### 4.6 Limitations and Strength of the Study

Limitations of the present study include the small sample size, the inclusion of participants from only one ethnicity group and a single examiner performing and analysing US scans. Furthermore, transducer choice or artefacts may have influenced the quality of the US images (Cumming et al. 2024), making it particularly challenging to define the edge of the ossification centre and the distal end of the epiphysis in metacarpal bones using the method developed by Wan et al. (Wan et al. 2019).

Another limitation is the absence of anthropometric data at the time of US measurements, which hinders retrospective analysis and limits the ability to compare the validity of the US method with other noninvasive approaches. This highlights the need to combine multiple assessment methods to improve the accuracy of maturity estimates (Leyhr et al. 2020).

Additionally, the potential effects of repetitive impacts and sport‐specific behaviours on bone development should be considered (Kannus et al. 1995; Haapasalo et al. 2000). As such, sports involving repeated impacts to a dominant hand often result in a unilateral higher bone density. In the context of soccer, goalkeepers are expected to have higher bone density in their upper extremities compared to field players due to repeated impacts from catching and diving for the ball (Kannus et al. 1995; Haapasalo et al. 2000).

Furthermore, the pre‐selected sample of goalkeepers is shifted towards biologically older players, as height and other maturity‐related characteristics are critical for goalkeeper performance. While this pre‐selected sample represents a limitation for generalisation to populations with an even distribution of BA, it accurately reflects the real‐world context of youth soccer goalkeepers (Sierra‐Díaz et al. 2017). On the other hand, the inclusion of goalkeepers provides valuable insights to a generally underrepresented cohort in research due to their low number compared to field players. Additionally, all participants were around PHV, which is where the greatest differences in BA occur, and thus is a particularly important phase for talent development and selection processes. Finally, newly developed methods, such as quantitative US should be considered, as they show promising results (Cumming et al. 2024; Rachmiel et al. 2017; Utczas et al. 2017; Ruf et al. 2021).

### 4.7 Future Directions

Future studies should reassess the validity of US, considering different body regions and potential operator dependency, by including multiple US examiners. The establishment of standardised parameters for US measurements (i.e., resolution, transducer positioning and angulation) and the exploration of combined methods (i.e., skeletal age and anthropometric measurements) using different body regions (i.e., hand, knee and elbow) should be investigated (Leyhr et al. 2020; Rüeger et al. 2022). Therefore, potential differences in bone development between goalkeepers and field players would be interesting. Future studies should include multiple raters to assess the validity and reliability of the conventional US method developed by Wan et al. (Wan et al. 2019), which recent studies have suggested is reliable (Lv et al. 2023; Hutmacher et al. 2024). Optimising transducer settings and implementing a standardised protocol could improve accuracy, precision and reliability (Contreras Ortiz, Chiu, and Fox 2012). In addition, to account for gender‐specific variations in biological development, future studies should include female participants (Cumming et al. 2024).

## 5 Conclusion

In summary, our pilot study has shown that using US‐derived OssR did not deliver valid results compared to X‐ray when determining BA in youth soccer goalkeepers. This discrepancy was emphasised by poor agreement in BA‐CA correlations and a lack of agreement between X‐ray and conventional US measurements. Conventional US measurements overestimated BA in younger participants and underestimated BA in older participants. Furthermore, the observed range of difference in estimated BA between X‐ray and conventional US measurements, discrepancies reaching up to 3.20 years, further highlights the necessity of accurate BA measurements, especially in sports settings where accurate talent identification and development are crucial. As a pilot investigation, this study provides an important foundation for future research to refine and improve US‐based methods for applications in sports.

## Author Contributions

Conceptualisation: C.W., J.D.B., D.‐P.B. and M.R.; Data acquisition: C.W. and J.D.B.; Data analysis: C.W.; Visualisation: C.W.; Writing – original draft preparation: C.W.; Writing – review and editing: M.R. and D.‐P.B.; Supervision: J.D.B., D.‐P.B. and M.R.; Project administration: D.‐P.B. and M.R.

## Conflicts of Interest

The authors declare no conflicts of interest.

## Data Availability

The datasets generated and/or analysed during this study are available in the OSF, https://osf.io/.

## Appendix

This appendix contains supplementary figures illustrating the imaging methods used in this study, including X‐ray and ultrasound scans of the left hand‐wrist, as well as a validation process diagram for determining biological age.
